# p53 coordinates base excision repair to prevent genomic instability

**DOI:** 10.1093/nar/gkw015

**Published:** 2016-01-14

**Authors:** Mattia Poletto, Arnaud J. Legrand, Sally C. Fletcher, Grigory L. Dianov

**Affiliations:** 1 CRUK & MRC Oxford Institute for Radiation Oncology, University of Oxford, Department of Oncology, Old Road Campus Research Building, OX37DQ Oxford, UK; 2 Institute of Cytology and Genetics, Siberian Branch of the Russian Academy of Sciences, Lavrenteva 10, 630090 Novosibirsk, Russia

## Abstract

DNA constantly undergoes chemical modification due to endogenous and exogenous mutagens. The DNA base excision repair (BER) pathway is the frontline mechanism handling the majority of these lesions, and primarily involves a DNA incision and subsequent resealing step. It is imperative that these processes are extremely well-coordinated as unrepaired DNA single strand breaks (SSBs) can be converted to DNA double strand breaks during replication thus triggering genomic instability. However, the mechanism(s) governing the BER process are poorly understood. Here we show that accumulation of unrepaired SSBs triggers a p53/Sp1-dependent downregulation of APE1, the endonuclease responsible for the DNA incision during BER. Importantly, we demonstrate that impaired p53 function, a characteristic of many cancers, leads to a failure of the BER coordination mechanism, overexpression of APE1, accumulation of DNA strand breaks and results in genomic instability. Our data provide evidence for a previously unrecognized mechanism for coordination of BER by p53, and its dysfunction in p53-inactivated cells.

## INTRODUCTION

Genomic DNA is inherently unstable due to its intrinsic chemical nature. It is estimated that as many as 10 000 DNA lesions/cell/day can arise under physiological conditions (1). If unrepaired, accumulation of these lesions results in mutations and leads to genomic instability, which is a hallmark of cancer cells (2). The DNA base excision repair (BER) pathway is a frontline mechanism preventing genomic instability, as it contributes to cell defence against most endogenous and exogenous sources of genotoxic lesions. BER is responsible for the elimination of base alterations (e.g. oxidation, alkylation) and DNA single strand breaks (SSBs); the latter can arise either spontaneously, or as a consequence of BER processing of damaged DNA bases (3). BER on base lesions is initiated by a damage-specific DNA glycosylase, which removes the aberrant base. The resulting abasic site (AP-site) is then processed by an apurinic/apyrimidinic endonuclease, which cleaves the phosphodiester bond 5′ to the baseless site, generating a SSB. In mammalian cells apurinic/apyrimidinic endonuclease 1 (APE1) accounts for the majority of the AP-site cleavage activity (4,5) and is therefore a crucial enzyme for mammalian DNA repair. At the same time, however, this makes APE1 responsible for the generation of most cellular SSBs occurring as BER by-products (6). In canonical BER, the resulting SSB is eventually sealed by a protein complex containing DNA polymerase β, X-ray repair cross-complementing protein 1 (XRCC1) and DNA ligase IIIα (3), where XRCC1 acts as a scaffold protein to coordinate the formation and ultimately the stability of the DNA polymerase β-XRCC1–DNA ligase IIIα complex on SSBs (7).

To minimize accumulation of SSBs during BER, the pathway likely requires strict control. In particular, there should be careful coordination between the level of APE1, which generates SSBs, and the downstream BER enzymes responsible for their resolution. This coordination becomes extremely important in the case of both aberrant expression of BER proteins (either increased or decreased) and of polymorphisms leading to lower or higher enzymatic activity of individual BER components. Importantly, these events have been extensively linked to genomic instability without understanding the underlying mechanism (8–11). Overexpression of APE1 has been observed in many cancer types, showing correlation with increased aggressiveness and therapy resistance (reviewed in (12,13)). Moreover, increased APE1 levels are known to generate genomic instability (14,15); possibly due to the lack of BER control and excessive generation of SSBs. Yet, the exact mechanism(s) modulating steady-state levels of BER proteins in order to promote BER coordination still remain elusive.

Here we hypothesized that APE1 amount might be regulated by the cellular load of persistently unrepaired DNA strand breaks. Consistent with this idea, recent observations have highlighted how other core BER components (i.e. Pol β, XRCC1 and DNA ligase IIIα) are regulated in a DNA damage load-dependent manner (11). In this work we provide evidence that p53 is central to BER coordination. We show that adjustments in APE1 expression occur at the transcriptional level, through a mechanism relying on p53-dependent destabilization of the transcription factor Sp1. Crucially, impairment of p53 function in cancer cells negatively affects the feedback response regulating APE1, leading to overproduction of APE1 and uncontrolled genomic instability.

Our work identifies a mechanism coordinating BER in normal cells, and explains how loss of p53 function in combination with impaired BER, leads to genomic instability.

## MATERIALS AND METHODS

### Cell culture and chemicals

Normal human fibroblasts TIG-1, WI38 and MRC5, as well as SV-40 transformed WI38 fibroblasts were from the Coriell Institute Cell Repository. LIMM-NBE1 cells were described in (16). All cells were cultured in DMEM (Invitrogen) supplemented with 15% foetal bovine serum at 37°C in a humidified atmosphere with 5% CO_2_. APE1 inhibitor III was from Calbiochem, AR03 was from Axon Medchem, PARP1 (ABT-888) and Sp1 (mithramycin A) inhibitors were from Enzo Life Sciences.

### Western blotting and antibodies

Whole cell extracts for western blotting were prepared as described previously (17). Proteins were separated by sodium dodecyl sulphate-polyacrylamide gel electrophoresis and transferred onto Immobilon-FL PVDF membranes (Millipore) according to standard procedures. Membranes were probed with the following antibodies: APE1 (Novus Biologicals, NB100–101), XRCC1 (Thermo Scientific, MS-1393-P0), α-actin (Abcam, ab6276), p21 (Cell Signaling Technology, 2947), p53 (Santa Cruz, sc-126), PAR (Trevigen, 4335-AMC-050), histone H2A.X phosphorylated at Ser139 (γH2AX, 05–636, Millipore), Sp1 (Millipore, 07–645), histone H3 (Cell Signaling Technology, 4499). Secondary antibodies conjugated with Alexa Fluor 680 (Molecular Probes) and IRDye^®^ 800 (Rockland) fluorescent dyes were used. Detection and quantification was carried out using an Odyssey image analysis system (Li-Cor Biosciences).

### RNAi and plasmid transfection

siRNA transfections were carried out using Lipofectamine RNAiMAX reagent (Invitrogen) according to the manufacturer's protocol. Unless otherwise indicated, cells were analysed 72 h after transfection. All siRNAs were used at a working concentration of 30 nM, with the exception of APE1 siRNA #1, which was used at 60 nM. siRNA oligonucleotides were from Eurogentec; a detailed list of the sequences can be found in the supplementary information. Control transfections were carried out using either a GFP-targeting siRNA, or a negative control siRNA (Eurogentec, SR-CL000-005). Plasmid transfections were carried out using the Viromer^®^ YELLOW reagent (Lipocalyx) as per manufacturer's indications. Cells were analysed 48 h after transfection, unless otherwise indicated. p53 encoding plasmids were previously described (18).

### Luciferase assays

Luciferase assays were carried out using a vector containing the APE1 promoter region spanning 4 kb upstream the *APEX1* gene (19). To assess the activity of the APE1 promoter, cells were first treated with the indicated siRNA. Transfection of the APE1 promoter plasmid was carried out 36 h after siRNA delivery and luciferase activity was measured 48 h post-transfection using a Dual-Glo^®^ Luciferase Assay System (Promega). Signal from a co-transfected pCMV-RL plasmid was used to normalize for transfection efficiency.

### Quantitative RT-PCR

Total RNA was extracted using the RNeasy kit (Qiagen) and cDNA was prepared using the SuperScript RT-PCR system (Invitrogen) as per manufacturer's indications. Quantitative RT-PCR was performed using SYBR^®^ Green PCR Master Mix (Life Technologies) according to the manufacturer's protocol. Reactions were carried out using a 7500 Fast Real-Time PCR System (Applied Biosystems). The comparative CT method was applied for quantification of gene expression; *GAPDH* or *B2M* were used as housekeeping genes. A list of the primers can be found in the Supplementary Information.

### Comet assays

Cells were harvested by trypsinization and analysed by alkaline and neutral Comet assays as described elsewhere (20,21).

### Chromatin immuno-precipitation (ChIP)

Chromatin immuno-precipitation (ChIP) assay was performed after cross-linking cells with 1.5% formaldehyde in culture medium for 15 min at room temperature; formaldehyde was quenched with 125 mM glycine and cells were lysed in lysis buffer (50 mM Tris–HCl pH 8.1, 10 mM ethylenediaminetetraacetic acid (EDTA), 1% sodium dodecyl sulphate (SDS), completed with protease inhibitors) on ice for 10 min. Chromatin was sonicated to an average fragment size of 200–1000 bp and clarified by centrifugation at 16 100 *g*. Protein concentration was measured using the Bio-Rad Protein Assay (Bio-Rad) and an equal amount of protein was diluted 5-fold in dilution buffer (16.7 mM Tris–HCl pH 8.1, 1.2 mM EDTA, 0.01% SDS, 1.1% Triton X-100, 167 mM NaCl, completed with protease inhibitors). To decrease non-specific binding chromatin was pre-cleared using protein A/G magnetic beads (New England Biolabs) for 1 h at 4°C. Four μg of either non-specific IgG (SantaCruz Biotechnology) or Sp1 antibody were added to the supernatant and rotated end-over-end overnight at 4°C. Protein–DNA complexes were then pulled-down by incubation with protein A/G magnetic beads for 2 h at 4°C; note that beads were pre-saturated with 0.1 mg/ml salmon sperm DNA (Invitrogen) and 0.1 mg/ml bovine serum albumin (BSA) (Sigma). After extensive washing, protein–DNA complexes were eluted using 0.1 M NaHCO_3_, 1% SDS. Proteins were removed by incubation in 40 mM Tris–HCl pH 6.5, 200 mM NaCl, 10 mM EDTA, 40 ng/μl proteinase K (Sigma), 20 ng/μl RNase A (Qiagen) for 3 h at 55°C. Cross-linking was reversed by heating overnight at 65°C and DNA was extracted using a QIAquick PCR Purification Kit (Qiagen). qPCR analysis was carried out as detailed above using the following primer pair to amplify the *APEX1* promoter (−161 to −26): ChIP_for: 5′-GCTAAGCGTCTCCGTCAC-3′ and ChIP_rev: 5′-CCGAGCACAAAGAAGGGTGC-3′.

### Electrophoretic mobility shift assays (EMSA)

For electrophoretic mobility shift assays (EMSA) reactions, nuclear extracts were generated as previously described (14). Binding reactions were set up by incubating 10 μg of nuclear extract into 20 mM Tris–HCl pH 7.5, 100 mM KCl, 10 mM ZnSO_4_, 0.2% NP-40, 20% glycerol, 5 mM dithiothreitol (DTT), supplemented with 1 μg salmon sperm DNA (Invitrogen). Reactions were started by adding 50 nM of double stranded *APEX1* promoter probe (−177 to −141) obtained by annealing a 5′-IRDye^®^800-labelled oligonucleotide (5′-AGAGGAGGGAGGCGAGGCTAAGCGTCTCCGTCACGTGG-3′ (Integrated DNA Technologies)), with its complementary sequence. After incubation for 15 min at 37°C, samples were separated onto a native 6% PAGE gel at 150 V for 50 min and analysed on an image analysis system (Li-Cor Biosciences).

### High-throughput immuno-fluorescence assays

Cells for microscopy assays were seeded in 96-well microplates (Corning) and transfected with the indicated plasmids. For combined knockdown/overexpression experiments cells were first treated with the indicated siRNA into 10 cm dishes and transferred into 96-well plates before overexpression. Immuno-staining was carried out on-plate following standard procedures, briefly cells were fixed with paraformaldehyde (4% in phosphate buffered saline (PBS) for 15 min), permeabilized using Triton X-100 (0.2% in PBS for 10 min at 4°C) and saturated with 5% BSA in PBS for 1 h. Incubation with antibodies was carried out in 5% BSA–PBS supplemented with 0.01% Tween 20. Alexa Fluor 488- and Alexa Fluor 594-conjugated secondary antibodies (Invitrogen) were used for indirect detection of the antigens and Hoechst 33342 (Life Technologies) was used to visualize nuclei. Images were acquired using an IN Cell Analyzer 1000 Imaging System and data were analysed using the IN Cell Investigator Software (GE Healthcare Life Sciences).

### Statistical analyses

Statistical analyses were performed by using the two-tailed Student's *t*-test using either Microsoft Excel or SPSS. Sample size is indicated for each experiment.

## RESULTS

### Accumulation of unrepaired SSBs leads to downregulation of APE1 transcription

**Figure 1.**
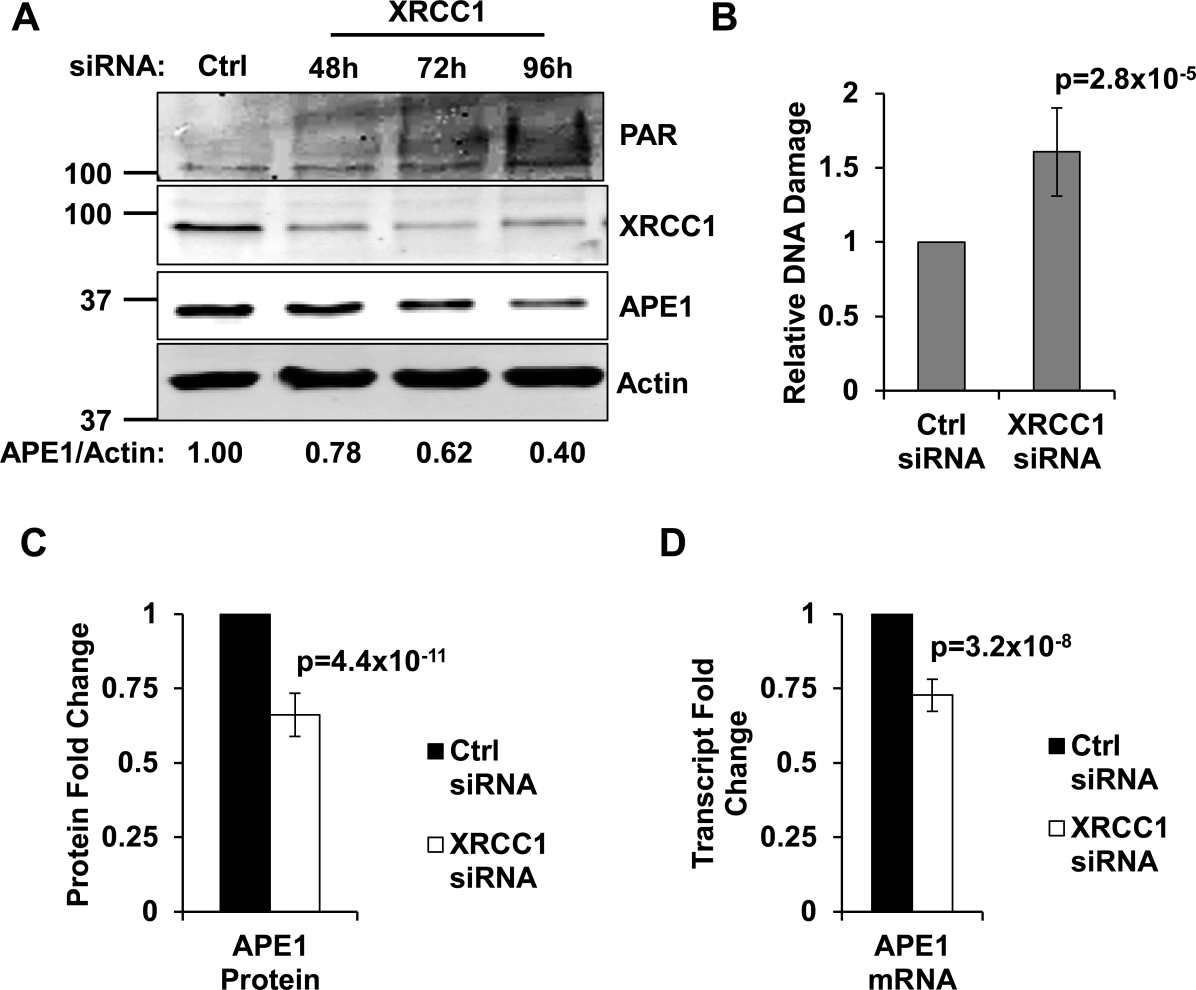
Persistent SSBs decrease APE1 levels by affecting its transcription. (**A**) Western blotting analysis on a representative XRCC1 knockdown time-course. TIG-1 cells were incubated with either a control siRNA (72 h), or a XRCC1-targeting siRNA, and harvested at the indicated time points. DNA damage accumulation is highlighted by PAR formation; APE1 is downregulated in a time-dependent manner. Actin was used as loading control. (**B**) Alkaline Comet assay on TIG-1 cells harvested 72 h after XRCC1 depletion shows accumulation of SSBs (*N* = 9). (**C**) Histogram showing the downregulation in APE1 protein amount 72 h after XRCC1 knockdown, as measured by western blotting (*N* = 10). (**D**) Histogram showing the downregulation in APE1 transcript level 72 h after XRCC1 depletion, as measured by qPCR (*N* = 7). Results depicted in histograms are presented as mean ± SD of the indicated number (*N*) of independent experiments.

An obvious way to coordinate the incision and ligation steps of BER is to control SSB production by regulating the amount/and or activity of APE1. BER is a very robust pathway: the vast majority of endogenous lesions are usually repaired within minutes (22). In order to be able to assess if unrepaired SSBs feed back to APE1, we forced TIG-1 normal human fibroblasts to accumulate endogenously generated SSBs by creating an artificial BER imbalance through transient knockdown of XRCC1. The XRCC1 knockdown phenotype in TIG-1 cells has been recently described by our laboratory; XRCC1-depleted fibroblasts have been shown to undergo a progressive accumulation of SSBs, enforcing a p53-dependent cell-cycle delay (23,10). As determined by increased poly(ADP-ribose) (PAR) synthesis (Figure 1A) and alkaline Comet assay (Figure 1B), XRCC1 depletion leads to an accumulation of SSBs. Consistent with our hypothesis, the time-dependent accumulation of SSBs correlates with a gradual decrease in APE1 amount (Figure 1A), resulting in a statistically significant reduction of APE1 protein amount (∼30% less) 72 h after XRCC1 depletion (Figure 1C). Downregulation of APE1 upon XRCC1 depletion was not restricted to TIG-1 cells, and was also observed in other normal diploid fibroblast cell lines as well as in immortalized epithelial cells (Supplementary Figure S1A). Importantly, consistent with the decreased APE1 amount, we observed a reduction in total AP-endonuclease activity in whole cell extracts obtained from XRCC1-depleted cells (Supplementary Figure S1B, S1C and SD), suggesting that even such a moderate adjustment in APE1 level is biologically relevant.

Similarly, we observed downregulation of APE1 in response to depletion of the DNA end-processing enzyme polynucleotide kinase 3′-phosphatase (PNKP, Supplementary Figure S2A), and upon knockdown (Supplementary Figure S2B) or inhibition (Supplementary Figure S2C) of poly(ADP-ribose) polymerase (PARP1), both enzymes involved in SSB repair. Since downregulation or inhibition of these proteins led to accumulation of DNA strand breaks, as measured by alkaline Comet assay (Supplementary Figure S2D), we concluded that APE1 downregulation is not solely linked to depletion of XRCC1, but is broadly triggered by unrepaired SSBs.

qPCR analyses showed that APE1 transcription was reduced 72 h after XRCC1 depletion (Figure 1D and Supplementary Figure S3). Although the measured gene expression changes were close to the confidence limit of the technique, the APE1 transcript reproducibly varied to a similar extent as that observed at the protein level. Additional analyses aimed at the measurement of transcriptional activity of the *APEX1* gene upon XRCC1 knockdown (see below) further validated this observation, confirming that APE1 is downregulated at the transcriptional level upon accumulation of unrepaired SSBs.

### APE1 downregulation upon SSB accumulation depends on p53 and Sp1

**Figure 2.**
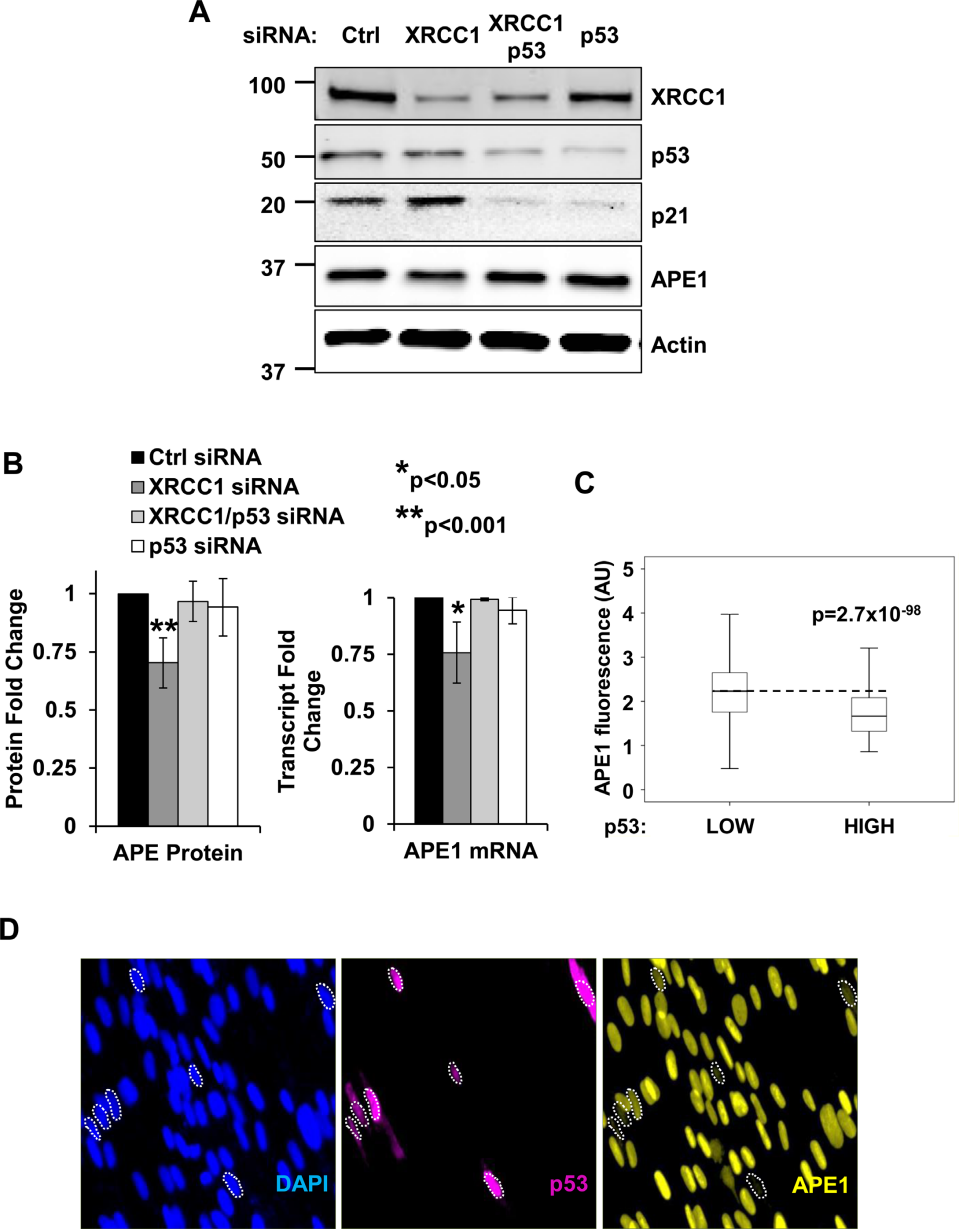
Downregulation of APE1 in response to BER unbalance is dependent of p53. (**A**) Representative western blotting analysis on TIG-1 cells depleted of XRCC1 and p53. APE1 is downregulated upon XRCC1 knockdown in a p53-dependent manner. Actin was used as loading control. (**B**) Left: histogram showing the quantification of APE1 protein amount in the experiment showed in panel A (*N* = 5). Right: histogram illustrating the amount of APE1 transcript upon combined XRCC1/p53 depletion, as measured through qPCR (*N* = 3). Results are expressed as mean ± SD of the indicated number (*N*) of independent experiments. (**C**) Boxplot showing the distribution of APE1 staining intensity (in arbitrary units) in p53 low versus p53 overexpressing cells. The dashed line highlights the median APE1 intensity in p53 low cells (*N* > 8000). (**D**) Representative high-throughput immuno-fluorescence pictures showing TIG-1 cells stained for APE1 (right panel) and p53 (middle panel) after transfection with a p53 expressing plasmid. Cells that downregulating APE1 in response to p53 overexpression are marked by a contour line.

The tumour suppressor p53 is a major transcription factor that determines cellular response upon DNA damage. XRCC1 depletion in normal diploid fibroblasts is known to elicit a p53-dependent cell-cycle delay (23,10), while a link between p53 and BER has been previously proposed by different groups (24,25). Earlier work has also suggested that the downregulation of APE1 in cells treated with the topoisomerase inhibitor camptothecin is p53 dependent although neither the mechanism triggering this process nor the biological role of APE1 modulation was clear (19). We therefore assessed if APE1 downregulation is triggered by unrepaired SSBs and whether this downregulation is p53 dependent. As illustrated in Figure 2A and in Supplementary Figure S4A, XRCC1 knockdown downregulates APE1 and activates p53, as measured by p21 induction. Furthermore, APE1 downregulation induced by XRCC1 depletion was completely rescued both at the protein (Figure 2A and B—left and Supplementary Figure S4B) and the transcriptional (Figure 2B—right) level by simultaneous p53 knockdown. These data demonstrate that unrepaired SSBs trigger APE1 downregulation in a p53-dependent manner.

To demonstrate direct involvement of p53 in APE1 downregulation we introduced exogenous p53 in TIG-1 fibroblasts and measured APE1 levels in p53 overexpressing cells. As plasmid transfection efficiency in normal diploid fibroblasts is fairly poor, we established a high-throughput immuno-fluorescence assay in order to compare the intensity of endogenous APE1 between untransfected and transfected cells. After confirming the ability of our assay to quantitatively measure APE1 downregulation upon XRCC1 depletion (Supplementary Figure S5A), we showed that overexpression of p53 results in a significant reduction in cellular APE1 levels (Figure 2C and D). Importantly, the specificity of our high-throughput immuno-fluorescence assay was demonstrated by the inability of overexpressed GFP to downregulate APE1 (Supplementary Figure S5B). Furthermore, p53 overexpression did not have any significant effect on the level of an unrelated target (i.e. histone H3, Supplementary Figure S5C). Taken together, these data show that accumulation of unrepaired SSBs activates p53, which in turn orchestrates the downregulation of APE1 expression.

**Figure 3.**
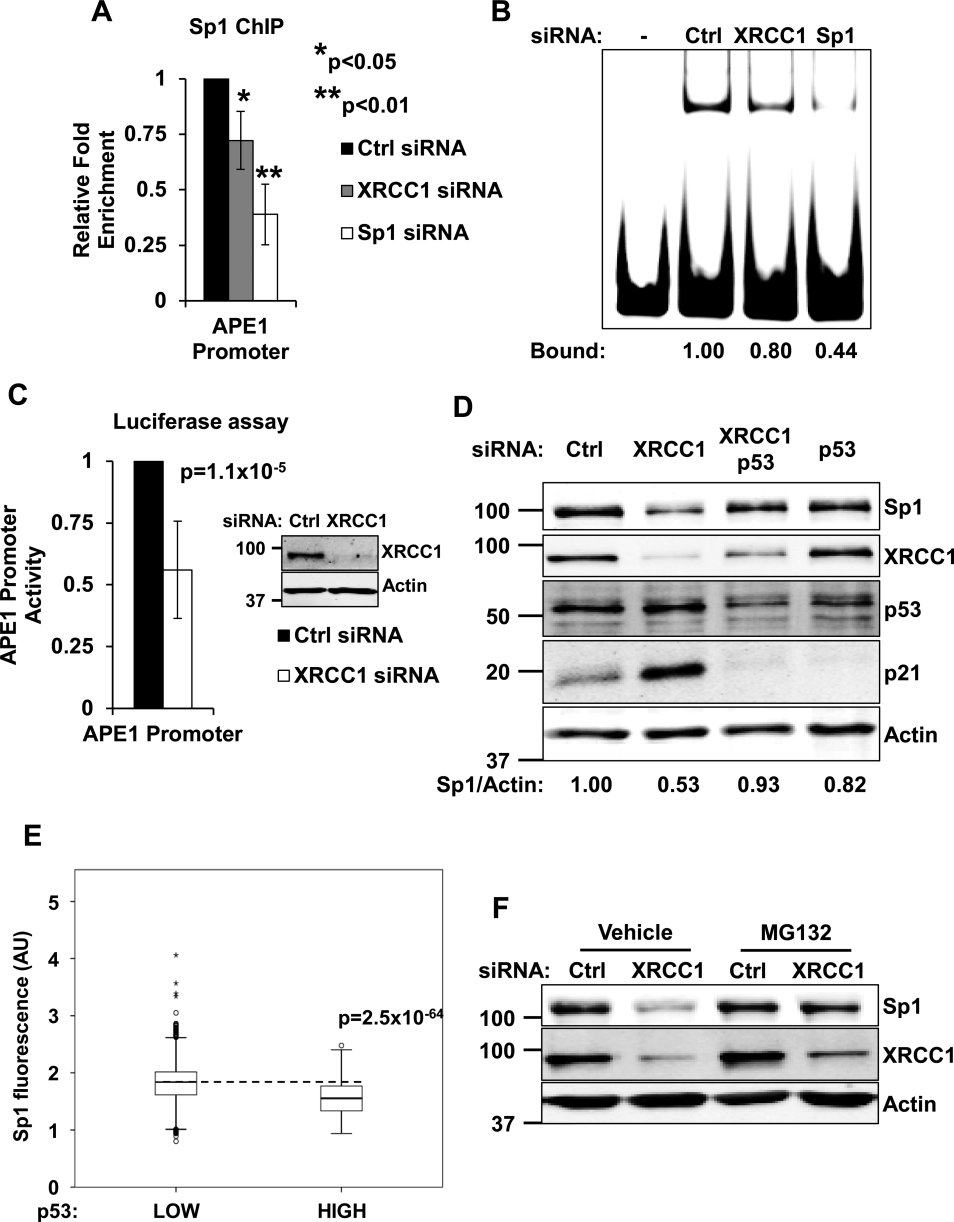
Accumulation of SSBs negatively affects Sp1 stability and activity towards the *APEX1* promoter. (**A**) Histogram displaying a ChIP analysis assessing Sp1 binding to *APEX1* proximal promoter. The assay was carried out using TIG-1 cells transfected with either a control siRNA, a XRCC1 siRNA or a Sp1-targeting siRNA (as positive control for signal reduction). Results are expressed as mean fold enrichment over unspecific IgG relative to control siRNA ± SD of three independent experiments. (**B**) Representative EMSA assay measuring Sp1 binding activity to *APEX1* proximal promoter. Cells were treated as described in panel A and nuclear extracts were used to assess Sp1 binding activity towards an *APEX1* promoter probe. Densitometric quantification of the DNA/protein complex is reported at the bottom of the picture. (**C**) Histogram illustrating the reduction in full length *APEX1* promoter activity in TIG-1 cells measured by luciferase assay upon XRCC1 knockdown. Results are expressed as mean relative promoter activity ± SD of nine independent experiments. Inset: western blotting showing a representative XRCC1 knockdown. (**D**) Representative western blotting analysis on TIG-1 cells depleted of XRCC1 and p53. Sp1 is downregulated upon XRCC1 knockdown in a p53-dependent manner. Actin was used as loading control. (**E**) Boxplot showing the distribution of Sp1 staining intensity (in arbitrary units) in p53 low versus p53 overexpressing cells. The dashed line highlights the median Sp1 intensity in p53 low cells (*N* > 8000). (**F**) Representative western blotting analysis showing rescue of Sp1 levels by proteasome inhibition (MG132 used 10 μM, 6 h) in a XRCC1-depleted background. Actin was used as loading control.

In order to gain further insights into APE1 downregulation in response to unrepaired SSBs, we investigated the mechanism by which p53 affects APE1 expression. Previous work demonstrated that p53 does not directly bind to the proximal promoter of the *APEX1* gene. Instead, p53 was proposed to inhibit APE1 expression by interfering with Sp1 binding activity, although the mechanism involved was not fully understood (19). We first verified that Sp1 indeed binds to the *APEX1* promoter and affects APE1 expression in normal fibroblasts. Sp1 depletion led to a reduction of APE1 protein and transcript amount (Supplementary Figure S6A and B); moreover, the *APEX1* promoter activity was greatly reduced in Sp1-depleted cells (Supplementary Figure S6C). In addition, cell treatment with the Sp1 inhibitor mithramycin A resulted in a rapid reduction of APE1 transcription (Supplementary Figure S6D). Finally, competition gel shift assays with an Sp1 consensus probe confirmed specific Sp1 binding at a region mapping between −177 and −141 of the *APEX1* promoter (Supplementary Figure S6E), consistent with previous findings (19,26). After confirming the contribution of Sp1 to APE1 expression in normal fibroblasts, we sought to assess the impact of unrepaired SSBs on Sp1-mediated transcription of the *APEX1* gene. In agreement with our data on transcription (Figure 1D), XRCC1-depleted cells showed decreased Sp1 occupancy on the proximal promoter of the *APEX1* gene, as measured both *in vivo* using ChIP assays (Figure 3A and Supplementary Figure S6F), and *in vitro* using EMSA assays (Figure 3B). Importantly, the reduction in Sp1 occupancy on the *APEX1* promoter in XRCC1 depleted cells (Figure 3A) functionally impacted transcription of the gene (Figure 1D) and correlated with decreased promoter activity, as measured by luciferase assays (Figure 3C).

Sp1 and p53 have been previously reported to cooperate to enhance (27), or to suppress (28) gene expression, in particular in response to DNA damage (29). In our experiments, we consistently observed a reduction in Sp1 protein amount upon XRCC1 depletion (Figure 3D) and interestingly, Sp1 fluctuations paralleled APE1 behaviour (Figure 2), changing in a p53-dependent manner (Figure 3D and Supplementary Figure S4B). In addition, overexpression of p53 decreased Sp1 protein levels, further confirming the key role of p53 in controlling Sp1 steady-state levels (Figure 3E). Curiously, despite the previous work proposed that p53 negatively affect Sp1 transcription (30), we did not detect any change in Sp1 expression levels upon XRCC1 depletion (Supplementary Figure S7). Proteasome inhibition, however, effectively rescued the Sp1 downregulation triggered by XRCC1 depletion (Figure 3F). We thus conclude that p53 controls *APEX1* gene transcription indirectly, through modulation of Sp1 stability.

### Impaired p53 function leads to an inability to coordinate BER and increases genomic instability

**Figure 4.**
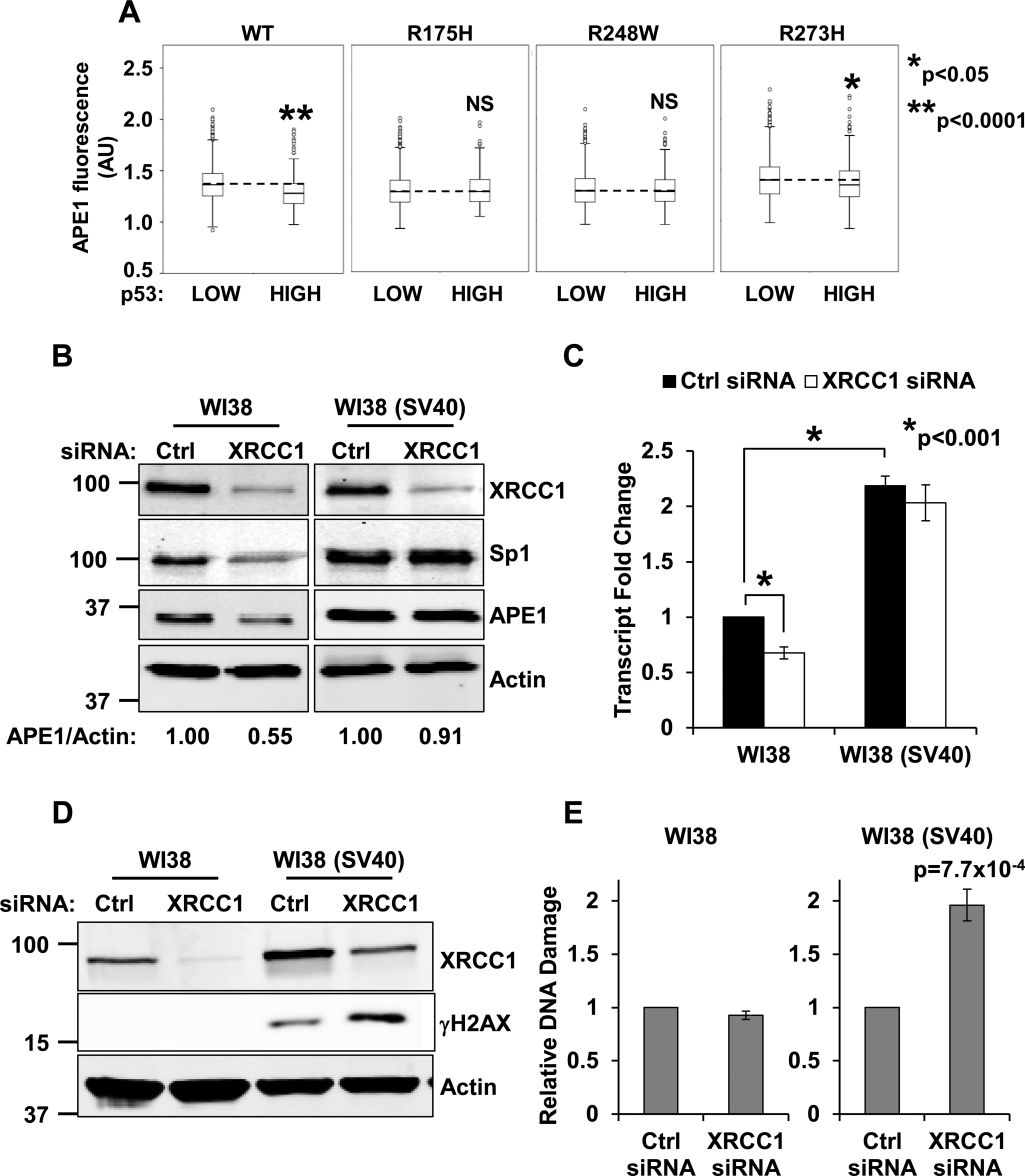
Defective p53 activity leads to failure of the BER coordination system. (**A**) High-throughput microscopy analysis of TIG-1 cells transfected with plasmids expressing wild-type or mutant p53. Boxplots showing the distribution of APE1 staining intensity (in arbitrary units) in p53 low versus p53 overexpressing cells. Each p53 mutant is reported on top of the relevant plot. The dashed line highlights the median APE1 intensity in p53 low cells (*N* > 5000). NS: not statistically significant at *P* < 0.05. (**B**) Representative western blotting analysis comparing WI38 and WI38 (SV40) cells upon transfection with the indicated siRNAs. Failure to downregulate Sp1 correlates with the inability to modulate APE1. Actin was used as loading control. (**C**) qPCR analysis on APE1 transcript in WI38 and WI38 (SV40) cells upon transfection with the indicated siRNAs. APE1 transcription is reduced in WI38 cells only. Note the higher transcript content in WI38 (SV40) cells. (**D**) Representative Western blotting analysis comparing WI38 and WI38 (SV40) cells upon transfection with the indicated siRNAs. Failure to modulate BER correlates with γH2AX staining in WI38 (SV40) cells; γH2AX increases further upon XRCC1 depletion. (**E**) Neutral Comet assay on WI38 and WI38 (SV40) fibroblasts shows accumulation of DSBs upon XRCC1-depletion in transformed cells only. Results are expressed as mean ± SD of three independent experiments.

The *TP53* gene is the most frequent target for mutation in cancer (31). Given the key role of p53 in the regulation of APE1 expression, we sought to assess whether BER coordination is affected when p53 function is impaired. To this aim, we overexpressed p53 ‘hot spot’ mutants (i.e. R175H, R248W and R273H) in TIG-1 cells. These proteins represent p53 missense mutations commonly found in human tumours and have been reported to affect p53 conformation (R175H), or DNA binding ability (R248W and R273H) (31). We found that although overexpression of wild-type p53 reduced APE1 expression (Figures 2C and 4A), overproduction of mutant proteins did not result in any significant reduction of APE1 staining (Figure 4A), suggesting that functional p53 is essential to modulate APE1 transcription. To further confirm this observation we compared the response of two isogenic cell lines (i.e. WI38 normal diploid fibroblasts and WI38 SV40-transformed fibroblasts—hereafter WI38 (SV40)) to accumulation of unrepaired SSBs. Consistent with the role of p53 in controlling APE1 steady-state level, we found that impairment of p53 function by SV40 transformation resulted in a deficiency in BER coordination upon XRCC1 depletion, as WI38 (SV40) cells were unable to downregulate Sp1 and APE1 protein levels (Figure 4B). In agreement with these data, XRCC1 depletion resulted in a downregulation of APE1 transcription in WI38, but not in WI38 (SV40) cells (Figure 4C). Notably, APE1 transcript amount was basally higher in WI38 (SV40) cells (Figure 4C), confirming the importance of p53 function for the overall modulation of APE1 expression. *In vitro* binding assays further confirmed these findings, showing a decreased Sp1 binding activity towards an *APEX1* promoter probe in cell extracts obtained from WI38 cells depleted for XRCC1, but not in WI38 (SV40) cells which, as expected, showed increased basal binding activity towards the *APEX1* promoter probe (Supplementary Figure S8A).

Imbalanced APE1 expression has been reported to generate genomic instability in transformed cells (14,15). We reasoned that the inability to modulate APE1 steady-state levels in WI38 (SV40), and the resulting overproduction of APE1 might generate uncontrolled genomic instability when BER coordination is required. Consistent with our hypothesis, WI38 (SV40) cells showed higher basal levels of genomic instability, as determined by increased phosphorylation of histone H2A.X at Ser139 (γH2AX) (Figure 4D). Induction of SSBs through XRCC1 depletion further exacerbated this phenotype (Figure 4D). Notably, γH2AX signal was undetectable in WI38 cells depleted of XRCC1, indicating that a proficient APE1 modulation mechanism in response to BER imbalance can indeed buffer the accumulation of unrepaired SSBs. These data were further substantiated by neutral Comet assays on the same isogenic pair of cell lines, which confirmed the accumulation of DNA double strand breaks (DSBs) in WI38 (SV40) cells only (Figure 4E). This phenomenon is likely to occur upon DNA replication and conversion of SSBs into DSBs, as WI38 (SV40) cells were unable to enforce a cell-cycle delay upon XRCC1 depletion (Supplementary Figure S8B).

**Figure 5.**
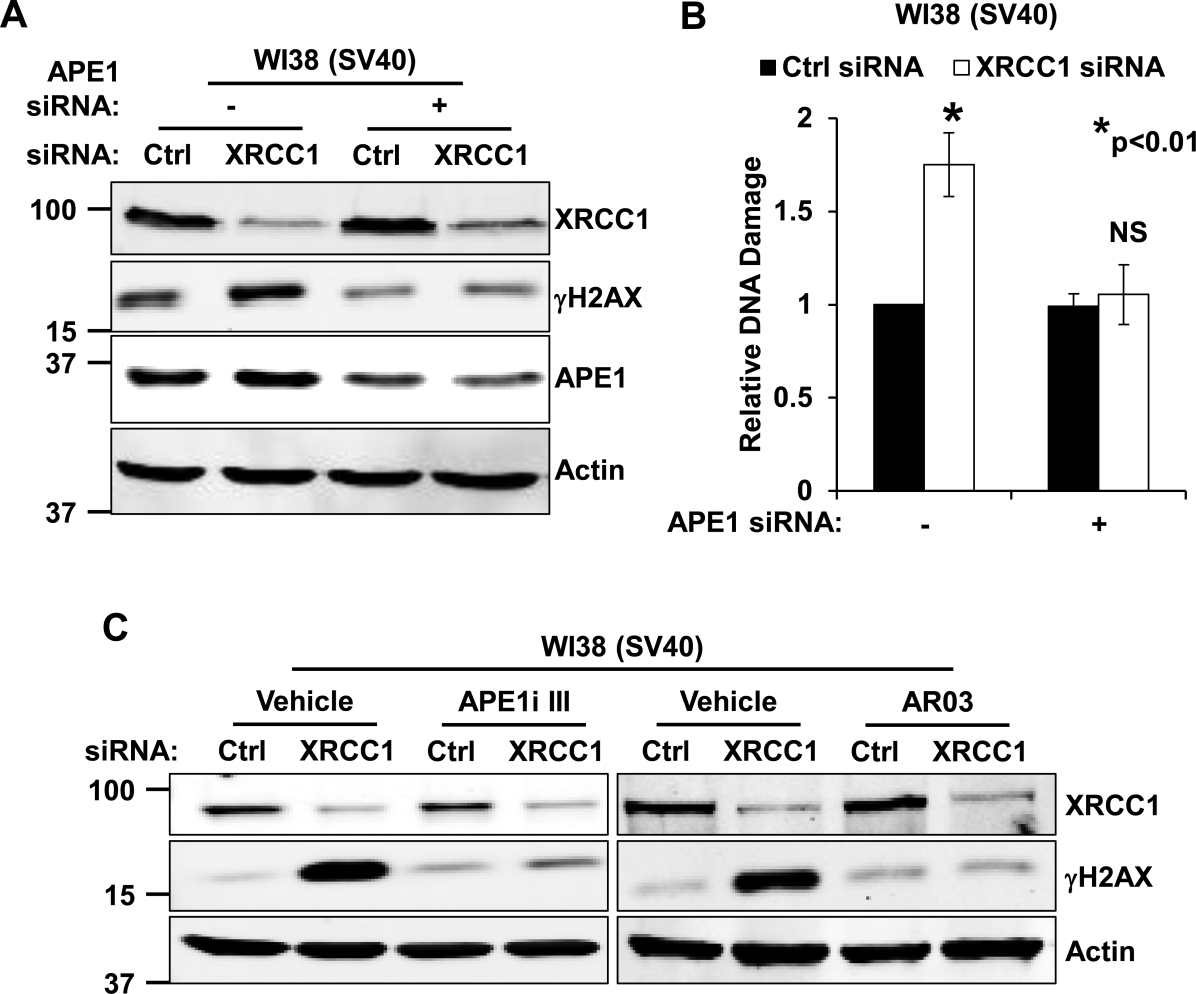
Excessive AP-endonuclease activity in cells with p53 impairment leads to accumulation of genomic instability. (**A**) Representative western blotting analysis on WI38 (SV40) cells shows rescue of γH2AX staining upon co-depletion of XRCC1 and APE1. (**B**) Neutral Comet assay on WI38 (SV40) fibroblasts shows accumulation of DSBs upon XRCC1-depletion and rescue after co-depletion of XRCC1 and APE1. Results are expressed as mean ± SD of three independent experiments. (**C**) Representative western blotting analysis on WI38 (SV40) cells shows rescue of γH2AX staining after depletion of XRCC1 in presence of APE1 inhibitor III (APE1i III, 5 μM for 24 h), or AR03 (2.5 μM for 24 h). Actin was used as loading control in all western blotting experiments.

To assess the contribution of APE1 to the genomic instability observed upon XRCC1 depletion in WI38 (SV40) cells, we simultaneously knocked down both APE1 and XRCC1. Western blotting (Figure 5A and Supplementary Figure S8C) and neutral Comet assay (Figure 5B) analyses confirmed that γH2AX and DSBs accumulation observed in WI38 (SV40) cells upon XRCC1 knockdown were dependent on APE1 expression. Generation of γH2AX in XRCC1 depleted WI38 (SV40) cells was also strongly reduced when APE1 endonuclease activity was inhibited with two different molecules (Figure 5C), confirming that the AP-site incision activity of the protein is responsible for the genomic instability observed upon BER imbalance.

Taken together, these data demonstrate that cells with impaired p53 signalling cannot properly coordinate BER. The inability to downregulate APE1 in response to SSB accumulation eventually leads to increased genomic instability, and this phenotype is mainly linked to an excess of AP-endonuclease activity.

## DISCUSSION

BER is a fundamental housekeeping DNA repair system that deals with the majority of endogenously generated DNA lesions. Taking into account the vast number of DNA lesions repaired by a human cell every day (1), and that SSBs are obligate intermediates during BER processing, it is becoming clear that lack of BER coordination can eventually result in accumulation of mutations and genomic instability.

Here we demonstrate that, in order to prevent genomic instability, cells coordinate the BER process by adjusting APE1 expression level through modulation of the stability of the Sp1 transcription factor. Although Sp1 has been proposed to contribute to the basal expression of the *APEX1* gene (19,26), its involvement in the regulation of BER in response to SSBs has not been addressed. Importantly, we show that p53 coordinates this process, although the mechanism leading to Sp1 degradation is presently unknown. The interaction between Sp1 and p53 has been previously described (28,32). As the p53/Sp1 association has been shown to increase upon DNA damage (29,33) it is possible to speculate that, in the absence of XRCC1, Sp1 stability might be indeed negatively affected via interaction with p53. p53 itself is known to be controlled by several post-translational modifications, including phosphorylation by the ataxia-telangiectasia mutated (ATM) protein kinase. Given that we have recently demonstrated ATM activation by SSBs (23), this kinase may represent a potential SSB sensor involved in BER coordination.

BER is fundamentally a robust system, endowed with a very efficient repair capability. Under physiological conditions, lesions generated endogenously, or even by short bursts of genotoxins (e.g. H_2_O_2_, MMS) are dealt with extremely quickly (22,17). To the best of our knowledge the pathway cannot be induced by genotoxic stress, as no changes in gene expression levels or protein stability are easily observed upon DNA damage. This is probably due to the extremely efficient buffering capacity of the pathway, when it operates correctly. Our work suggests that the mechanisms coordinating BER are triggered by adaptation of the pathway to a situation of persistent DNA damage that is manifested when BER is impaired (e.g. upon XRCC1 depletion). Such BER dysregulation is likely very common and can occur during physiological processes, such as tissue differentiation (34–36), or in the presence of polymorphisms in BER genes (37). In this work we generated an artificial BER imbalance by transient XRCC1 knockdown in normal fibroblasts. This allowed us to investigate the mechanisms involved in BER coordination. We conclude that in a condition of BER dysregulation p53 becomes extremely important not only for controlling DNA replication delay in response to DNA damage, but also for the coordination of the BER pathway. Importantly, our work also proves that impairment of p53 function in a background of BER imbalance can eventually lead to a mutator phenotype.

The positive correlation between APE1 expression levels, tumour aggressiveness and poor prognosis is well-established (reviewed in (12,13)). To the best of our knowledge, however, the mechanism underlying the failure of APE1 modulation in cancer has never been addressed before. Here, as a proof of concept, we use p53 mutant forms and we compare isogenic normal and SV40-transformed fibroblasts. Our data demonstrate that impairment of p53 function leads to overproduction of APE1. It is possible that similar mechanisms operate in cancer cells deficient for p53 function, thus explaining the APE1 overexpression pattern typical of many cancer types (12,38–41). However, we were not able to observe any modulation of APE1 expression in cancer cell lines bearing differential p53 status (e.g. HeLa, U2OS—data not shown). We thus conclude that the mechanism of regulation of APE1 might fully respond to endogenous DNA damage only in genetically stable cell lines (e.g. normal fibroblast or immortalized epithelial cells), but is strongly impaired in transformed cells, where genetic instability is already established.

Interestingly, our data would predict correlation between APE1 expression and p53 status in cancer cells. Indeed, inverse correlation between APE1 and wild-type p53 expression has been observed in cervical and non-small cell lung cancer (NSCLC) specimens (41,42). Recently, Cun and colleagues observed higher APE1 expression in association with p53 mutation in hepatocellular carcinoma (43). Conversely, while inverse correlation between p53 and APE1 expression was measured in head-and-neck cancer specimens, this did not seem to be related to the status of p53 (44). Interestingly, Wu *et al*. recently showed that positivity for the human papilloma virus protein E6, which inactivates p53, is correlated with higher cytoplasmic APE1 expression in NSCLC. Thus suggesting that inactivation of p53 function can indeed affect APE1 expression in humans (45). Overall, it seems likely that APE1 amount indeed correlates with p53 expression level, even though more targeted studies will clearly be needed to understand whether the status of p53 could affect APE1 expression in cancer.

In conclusion, our study uncovers a previously unrecognized role for p53 protein in coordinating the BER process and demonstrates that this mechanism is impaired in p53-inactivated cells.

## Supplementary Material

SUPPLEMENTARY DATA
