# Supplementary material for: p53 coordinates base excision repair to prevent genomic instability
Source: Nucleic Acids Res. 2016 Jan 14;44(7):3165–75. doi: 10.1093/nar/gkw015 (PMC4838360; doi:10.1093/nar/gkw015)
Supplement: SUPPLEMENTARY DATA [file supp_44_7_3165__index.html]

p53 coordinates base excision repair to prevent genomic instability — p53 coordinates base excision repair to prevent genomic instability — p53 coordinates base excision repair to prevent genomic instability — SUPPLEMENTARY DATA 

# p53 coordinates base excision repair to prevent genomic instability

## SUPPLEMENTARY DATA

- SUPPLEMENTARY DATA
