# Supplementary material for: p53 coordinates base excision repair to prevent genomic instability
Source: Nucleic Acids Res. 2016 Jan 14;44(7):3165–75. doi: 10.1093/nar/gkw015 (PMC4838360; doi:10.1093/nar/gkw015)
Supplement: SUPPLEMENTARY DATA [file supp_gkw015_nar-02957-d-2015-File003.docx]

**p53 coordinates base excision repair to prevent genomic instability**

Mattia Poletto^1^, Arnaud J. Legrand^1^, Sally C. Fletcher^1^ and Grigory L. Dianov^1,2*^

^1^CRUK & MRC Oxford Institute for Radiation Oncology, University of Oxford, Department of Oncology, Old Road Campus Research Building, OX37DQ Oxford, UK

^2^Institute of Cytology and Genetics, Siberian Branch of the Russian Academy of Sciences, Lavrenteva 10, 630090 Novosibirsk, Russia

^*^To whom correspondence should be addressed: Tel: +44 1865 617325, Fax: +44 1865 617355, email: grigory.dianov@oncology.ox.ac.uk

**Supplementary Information**

The following file contains supplementary material for the paper “*p53 coordinates base excision repair to prevent genomic instability*”, by Poletto M. *et al*. This file is composed of:

- Supplementary methods
- Supplementary figures and relative supplementary figure legends (8 figures)
- Supplementary references

**Supplementary Methods**

**siRNA sequences used in this study.**

| **Target** | **Sequence (3’ to 5’)** | **Reference** |
| --- | --- | --- |
| **XRCC1 #1** | AGGGAAGAGGAAGUUGGAU | [1] |
| **XRCC1 #2** | GGAAGAUAUAGACAUUGAG | [2] |
| **XRCC1 #3** | GCUUGAGUUUUGUACGGUU | [3] |
| **p53 #1** | AAGACUCCAGUGGUAAUCUAC | [4] |
| **p53 #2** | GCAUGAACCGGAGGCCCAU | [5] |
| **Sp1 #1** | AACAGCGUUUCUGCAGCUACC | [6] |
| **Sp1 #2** | UAUUUGACCAGAACCAUCC | - |
| **APE1 #1** | AAUGACAAAGAGGCAGCAGG  Note: used at 60 nM | [7] |
| **APE1 #2** | UACUCCAGUCGUACCAGACCU | [8] |
| **GFP** | GCUGACCCUGAAGUUCAUCUU | [9] |

**Quantitative RT-PCR primers used in this study.**

| **Target** | **Sequence (3’ to 5’)** |
| --- | --- |
| **APE1** | For: CGGACAAGGAAGGGTACAGT  Rev: CAAATTCAGCCACAATCACC |
| **Sp1** | For: CTATAGCAAATGCCCCAGGT  Rev: TCCACCTGCTGTGTCATCAT |
| **B2M** | For: ATGTCTCGCTCCGTGGCCTTA  Rev: ATCTTGGGCTGTGACAAAGTC |
| **GAPDH** | For: AGCCACATCGCTCAGACAC  Rev: GCCCAATACGACCAAATCC |

**AP-endonuclease assays.** APE1 endonuclease activity in whole cell extract was monitored as already described [10], with minor modifications. Enzymatic reactions were carried out in a buffer containing 25 mM Tris-HCl pH 7.4, 100 mM KCl, 1 mM MgCl_2_ and 1 mM DTT. Reactions were started by adding 50 nM of double stranded abasic DNA substrate (obtained by annealing a 5’-IRDye^®^800-labelled oligonucleotide 5’-AATTCACCGGTACGFTCTAGAATTCG-3’ (Integrated DNA Technologies), where F indicates a tetrahydrofuran residue, with the complementary sequence 5’-CGAATTCTAGACCGTACCGGTGAATT-3’) and incubated at 37°C for the indicated time points. Reactions were halted by addition of formamide buffer (96% formamide, 10 mM EDTA, and traces of bromophenol blue), separated onto a 20% denaturing polyacrylamide gel and analysed on an image analysis system (Li-Cor Biosciences).

**Cell-cycle analysis.** Cells were harvested by trypsinisation and fixed with ice-cold 70% ethanol for 30 minutes. Cells were then incubated for 30 minutes at 37°C with RNAse A (100 μg/ml) in PBS and then stained with propidium iodide 10 μg/ml (Sigma). Samples were analysed on a Becton-Dickinson FACScan (BD Biosciences) and data were analysed using the Modfit LT software (Verity Software House).

**Supplementary Figures**

**
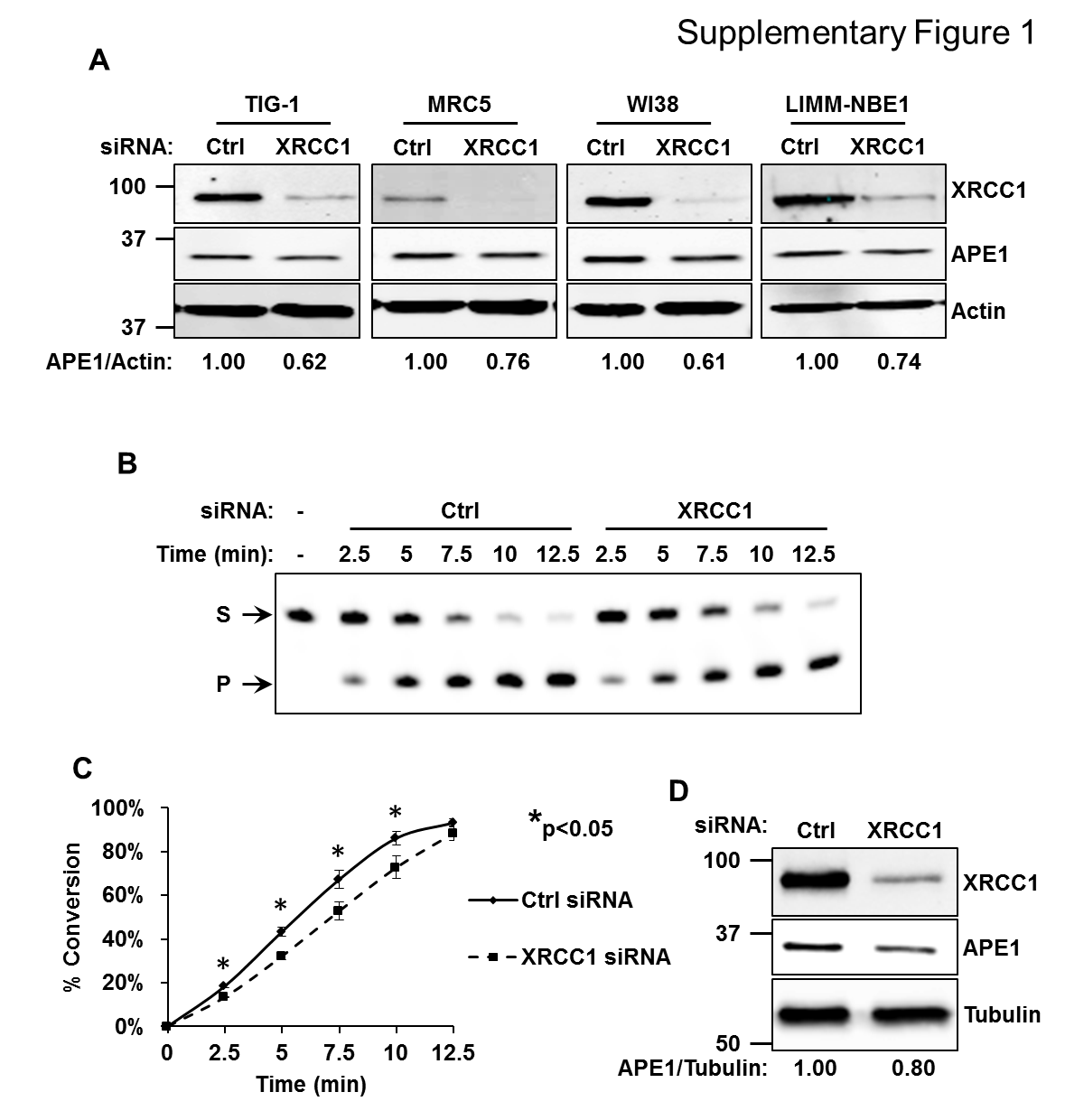
**

**Supplementary Fig. 1 – APE1 is downregulated upon XRCC1 depletion.**

**A.** Representative Western blotting analysis on different normal diploid fibroblast cell lines showing the downregulation of APE1 protein upon XRCC1 knockdown. Cell were harvested 72 hours after XRCC1 depletion and subjected to Western blotting. Actin was used as loading control. **B.** Representative picture of a typical AP-endonuclease activity assay. Equal amounts of whole cell extract obtained from either control siRNA- or XRCC1 siRNA-treated TIG-1 cells were incubated for the indicated amount of time with an oligonucleotide substrate containing an artificial AP-site. Activity was calculated as percentage of substrate (S) converted to product (P). **C.** AP-endonuclease activity curves obtained for control- and XRCC1-depleted cells; a moderate, but statistically significant reduction in AP-endonuclease activity is observed in XRCC1 knockdown cells. Results are expressed as mean ± SD of three independent experimental replicates. **D.** Western blotting analysis on the TIG-1 whole cell extracts compared in the endonuclease assays presented in panel B and C. Tubulin was used as loading control.


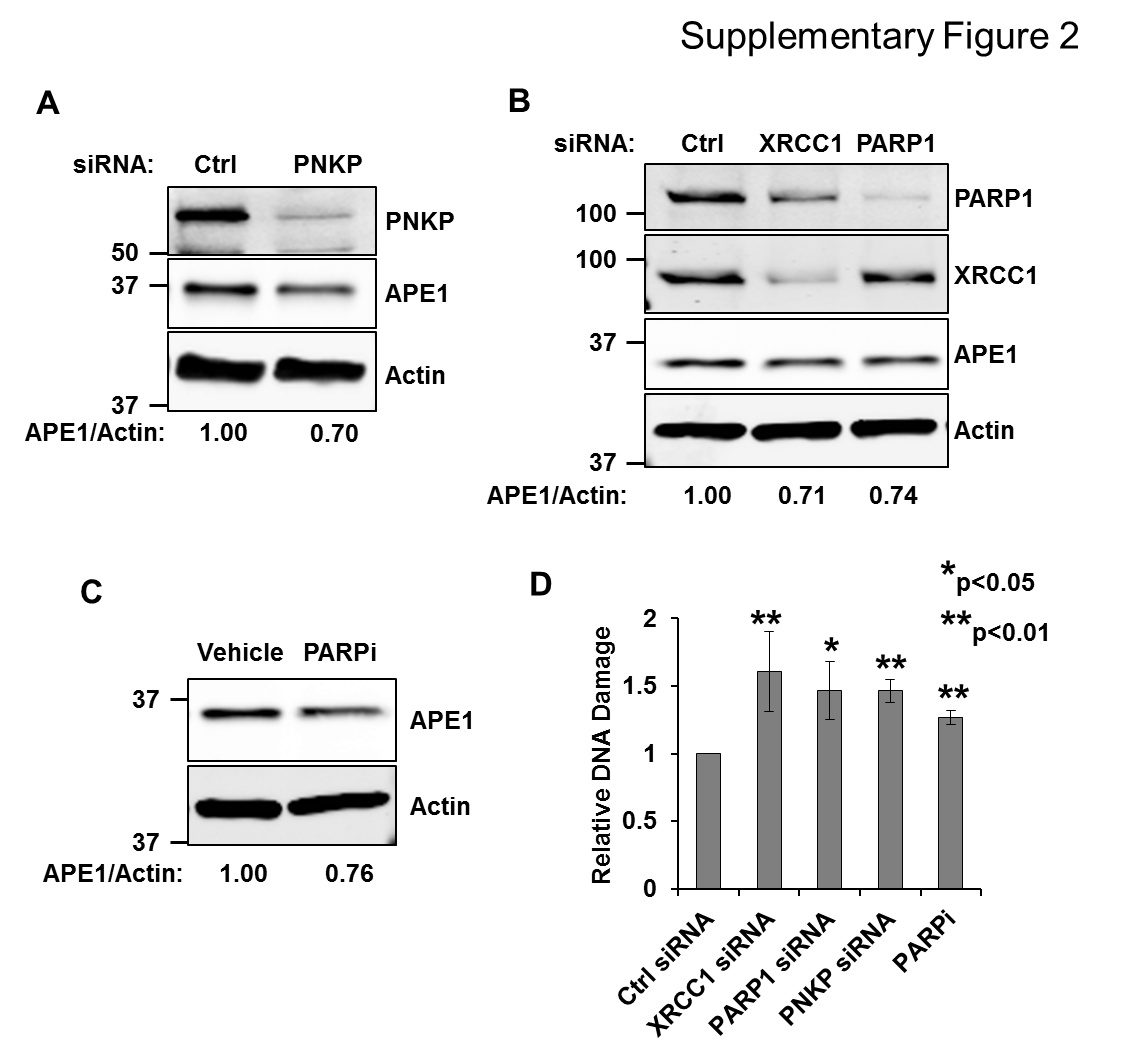


**Supplementary Fig. 2 – Persistent unrepaired strand breaks trigger APE1 downregulation.**

**A.** Representative Western blotting analysis showing the downregulation of APE1 upon PNKP knockdown. **B.** Representative Western blotting analysis comparing APE1 levels upon XRCC1 or PARP1 depletion in TIG-1 cells. **C.**  Representative Western blotting analysis comparing APE1 levels upon PARP inhibition. PARP inhibitor (PARPi) was used at 10 μM for 72 hours; fresh inhibitor was supplied every 24 hours. **D.** Alkaline Comet assay analysis on TIG-1 cells treated with the indicated siRNA or inhibitor. Results are expressed as mean ± SD of at least three independent experiments. For all Western blotting actin was used as loading control.


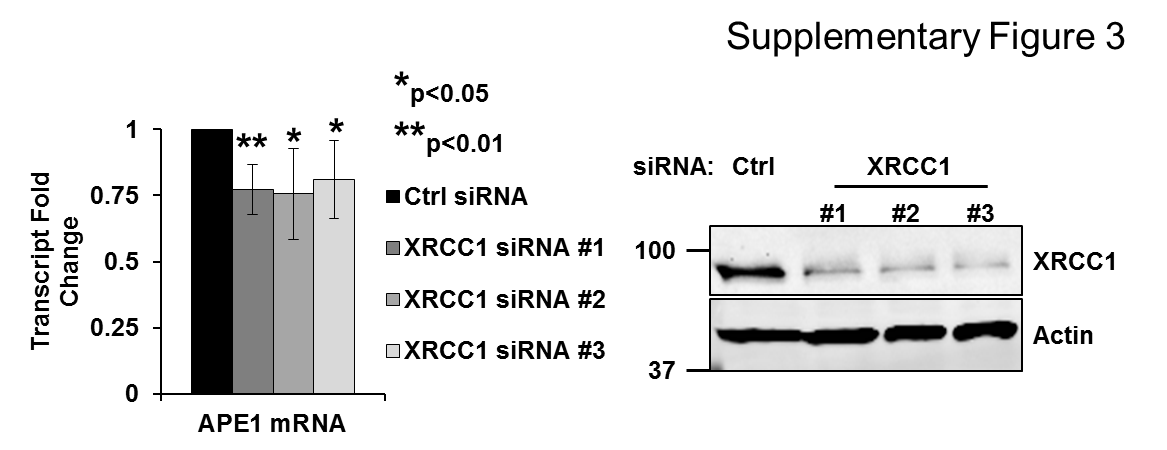


**Supplementary Fig. 3 – APE1 is regulated at the transcriptional level.**

*Left:* qPCR analysis on TIG-1 cells analysing APE1 transcript amount upon XRCC1 knockdown with three different siRNA sequences. APE1 transcription is consistently reduced with all the sequences tested. Results are expressed as mean ± SD of at least three independent experiments. *Right:* Western blotting analysis showing the reduction in XRCC1 protein level after depletion with the siRNA sequences used in the left panel. For all Western blotting actin was used as loading control.

**
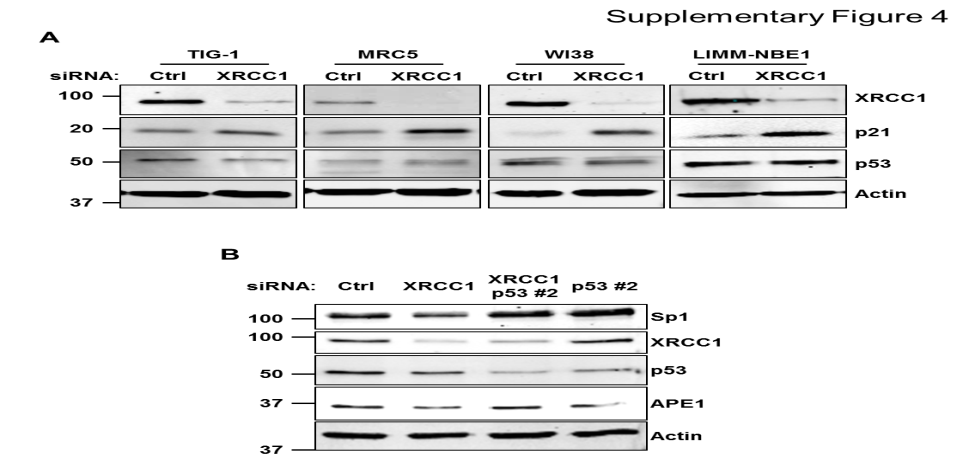
**

**Supplementary Fig. 4 – p53 is activated by XRCC1 depletion in different normal fibroblast cell lines.**

**A.** Representative Western blotting analysis on different normal diploid fibroblast cell lines showing activation (but not stabilisation) of p53 upon XRCC1 depletion, as assessed by p21 induction. Note that the pictures refer to the same experiment presented in Supplementary Fig. 1. Actin was used as loading control. **B.** Western blotting analysis on TIG-1 cells showing rescue of APE1 and Sp1 levels upon simultaneous XRCC1 and p53 depletion. The experiment was performed using p53 siRNA sequence #2.

**
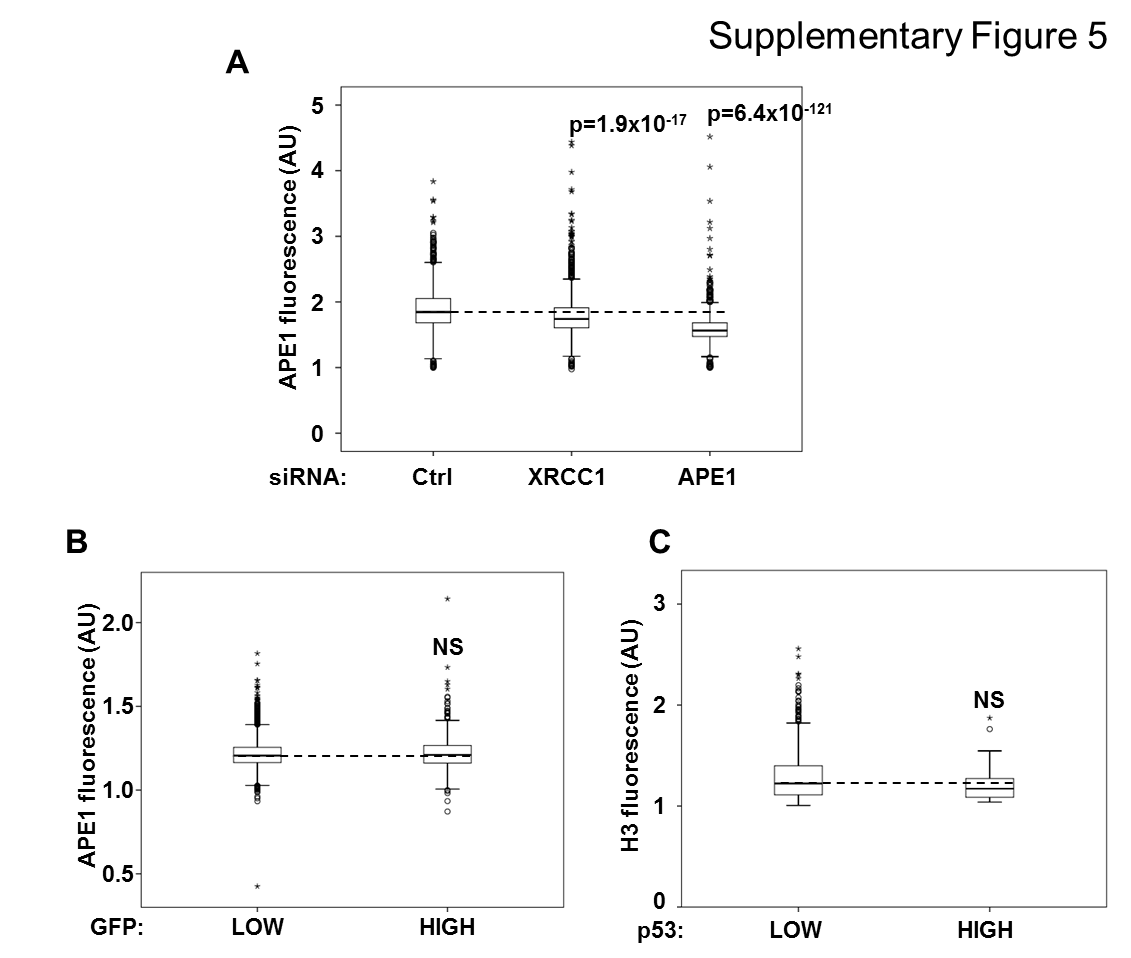
**

**Supplementary Fig. 5 – A high-throughput microscopy approach quantitatively detects APE1 downregulation in TIG-1 fibroblasts.**

**A.** Boxplot showing distribution of APE1 staining intensity (in arbitrary units) in TIG-1 cells transfected with either a control siRNA or depleted of XRCC1 or APE1. The dashed line highlights the median APE1 intensity in control cells. **B.** Boxplot showing distribution of APE1 staining intensity (in arbitrary units) in TIG-1 cells transfected with either an empty plasmid or a GFP-expressing plasmid. The dashed line highlights the median APE1 intensity in control cells. **C.** Boxplot showing distribution of histone H3 staining intensity (in arbitrary units) in TIG-1 cells overexpressing p53. The dashed line highlights the median H3 intensity in p53 low cells. (N>5,000). NS: not statistically significant at p<0.05.


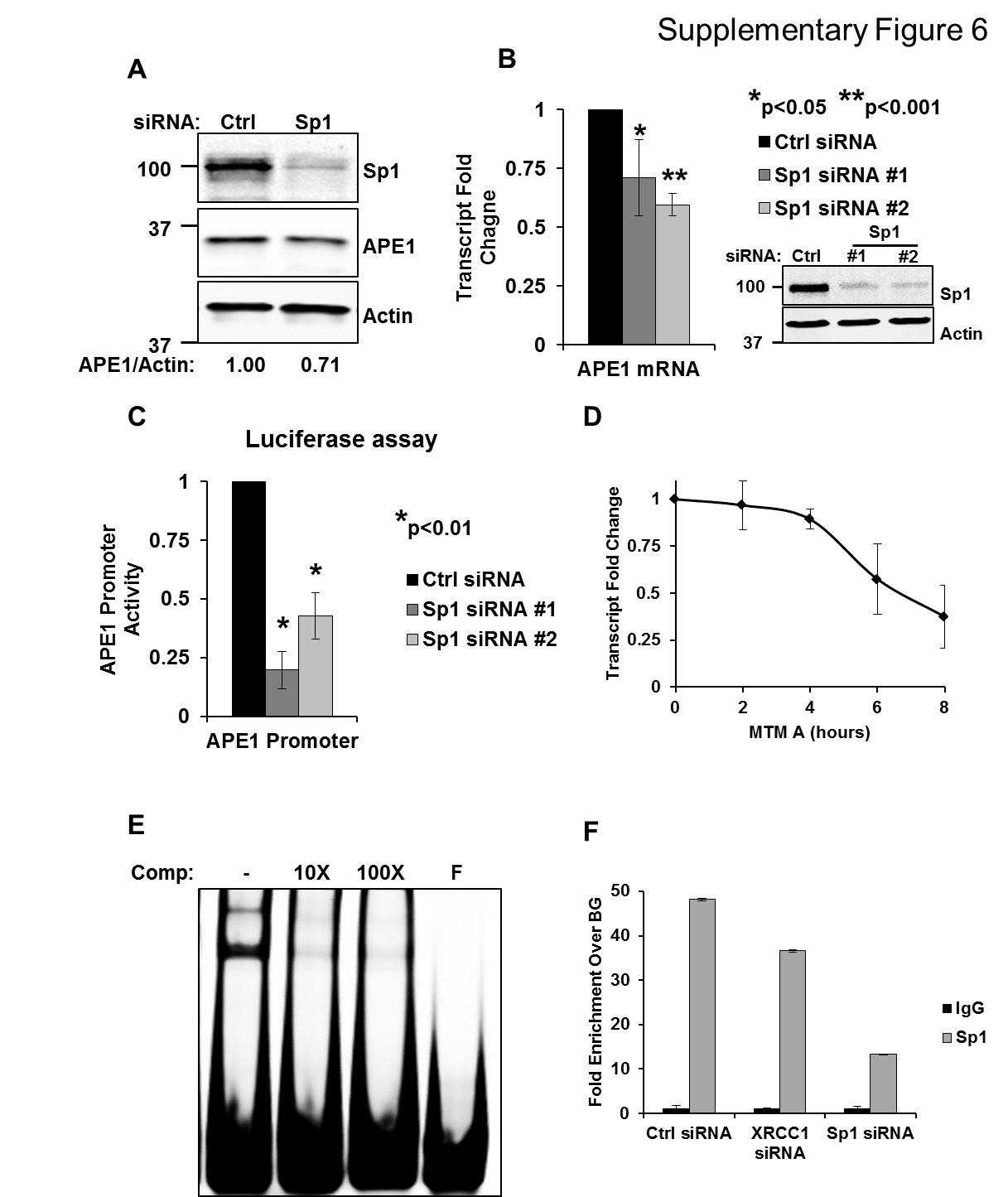


**Supplementary Fig. 6 – APE1 expression is modulated by Sp1.**

**A.** Representative Western blotting analysis showing APE1 downregulation upon Sp1 depletion in TIG-1 cells. Actin was used as loading control. **B.** qPCR analysis on Sp1-depleted TIG-1 cells shows the downregulation of APE1 transcription with two different Sp1 siRNAs (N=3). Sp1 knock-down efficiency is shown in the Western blotting inset. **C.** Luciferase assay showing the decrease in *APEX1* promoter activity upon Sp1 knockdown in TIG-1 cells (N=3). Two different siRNA sequences were used. **D.** qPCR analysis on TIG-1 fibroblasts challenged for the indicated amount of time with the Sp1 inhibitor Mithramycin A (MTM A, 1 μM). The graph shows the decrease in APE1 transcript level (N=3). **E.** Representative EMSA using nuclear extracts from TIG-1 cells co-incubated with an APEX*1* promoter probe. The picture shows the decrease in bound complexes upon competition with 10- or 100-fold excess of unlabelled Sp1 consensus sequence (5’- ATTCGATCGGGGCGGGGCGAGC-3’ [11]). F: free probe. **F.** Representative ChIP analysis on TIG-1 cells treated with the indicated siRNA. The histogram shows a typical ChIP experiment highlighting the fold enrichment in *APEX1* promoter over the background (unspecific IgG) after pull-down with a Sp1 antibody. The experiment reported here shows the raw fold enrichment from a single experiment run in duplicate. Normalised data for independent experiments are reported in Fig. 3A. Results are expressed as mean ± SD of the indicated number (N) of independent experiments.


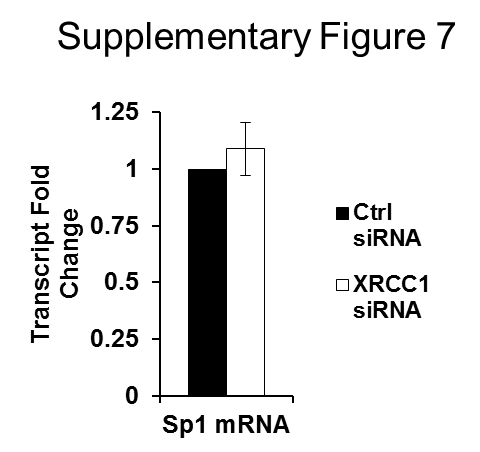


**Supplementary Fig. 7 – XRCC1 knockdown does not affect Sp1 transcription.**

qPCR analysis on TIG-1 cells shows no difference in Sp1 transcription upon XRCC1 depletion (N=5).


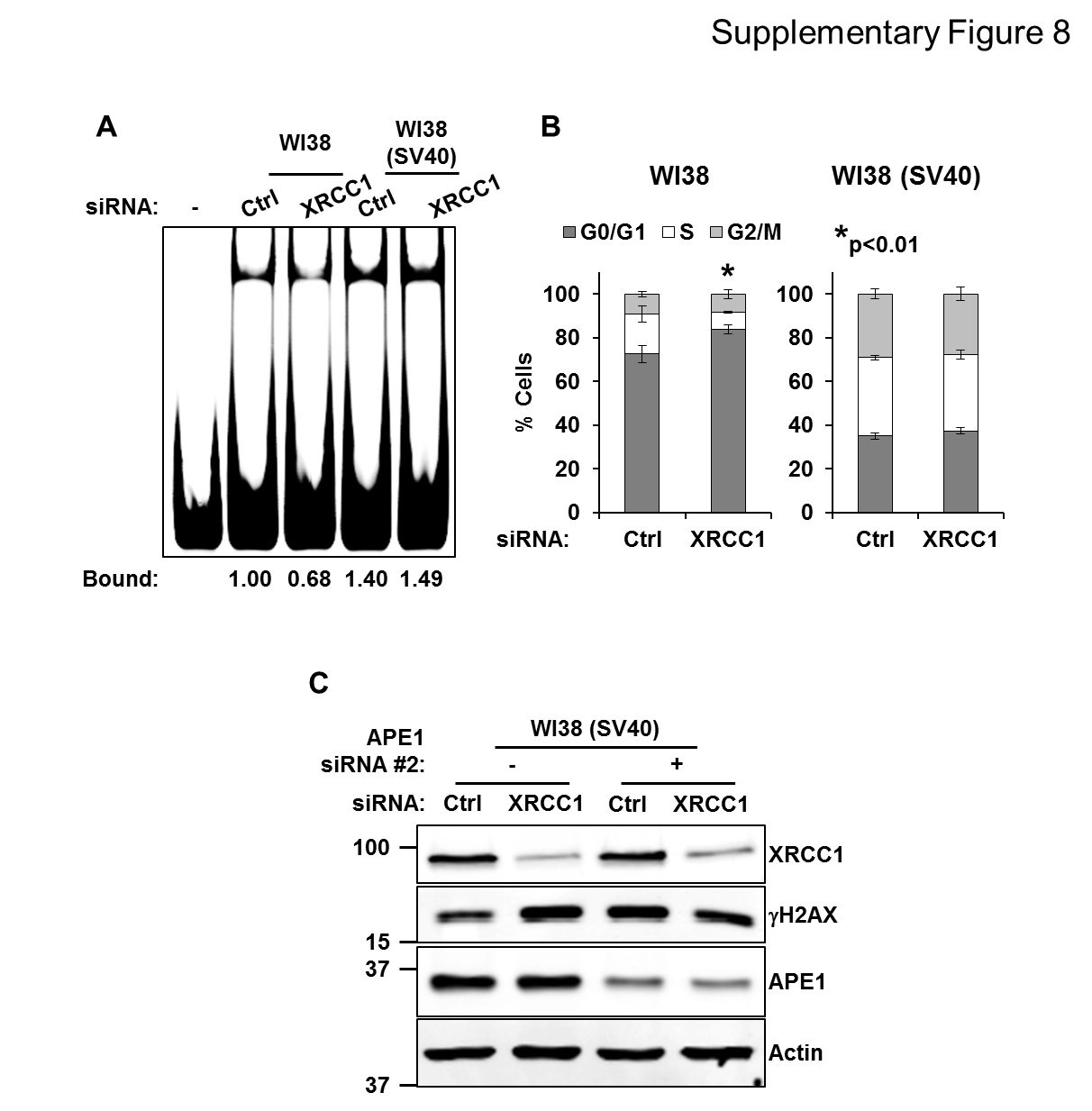


**Supplementary Fig. 8 – Impaired p53 function leads to defective Sp1 and cell-cycle response upon XRCC1 knockdown.**

**A.** Representative EMSA assay measuring Sp1 binding activity towards an APEX*1* promoter probe. WI38 and WI38 (SV40) cells were depleted of XRCC1 and equal amounts of nuclear extracts were used to assess Sp1 binding activity. Densitometric quantification of the DNA/protein complex is reported at the bottom of the picture. **B.** FACS analysis comparing WI38 and WI38 (SV40) cell-cycle profile upon XRCC1 depletion. While WI38 cells show marked G_0_/G_1_ delay, WI38 (SV40) cells fail to enforce cell-cycle arrest upon XRCC1 knock-down (N=3). Statistical analysis only refers to the difference in G_0_/G_1_ content between control and XRCC1 siRNAs. **C.** Representative Western blotting analysis on WI38 (SV40) cells upon co-depletion of XRCC1 and APE1, shows inability of cells to accumulate γH2AX further. The experiment was performed using the APE1 siRNA sequence #2. Note that this sequence is more effective than APE1 #1 in terms of residual APE1 knock-down (compare with Figure 5A, where twice as much APE1 siRNA #1 was used); this could explain the higher basal genomic instability observed in APE1-depleted cells with this sequence.

**Supplementary References**

1. Brem,R. and Hall,J. (2005) XRCC1 is required for DNA single-strand break repair in human cells. *Nucleic Acids Res.* **33,** 2512–2520.

2. Fan,J., Wilson,P.F., Wong,H.K., Urbin,S.S., Thompson,L.H. and Wilson,D.M.3rd. (2007) XRCC1 down-regulation in human cells leads to DNA-damaging agent hypersensitivity, elevated sister chromatid exchange, and reduced survival of BRCA2 mutant cells. *Environ. Mol. Mutagen.* **48,** 491-500.

3. Sultana,R., Abdel-Fatah,T., Abbotts,R., Hawkes,C., Albarakati,N., Seedhouse,C., Ball,G., Chan,S., Rakha,E.A., Ellis,I.O., *et al.* (2013) Targeting XRCC1 deficiency in breast cancer for personalized therapy. *Cancer Res.* 73, 1621-1634.

4. Zhu,W., Chen,Y. and Dutta,A. (2004) Rereplication by depletion of geminin is seen regardless of p53 status and activates a G2/M checkpoint. *Mol. Cell. Biol.* **24,** 7140–7150.

5. Di Pietro,A., Koster,R., Boersma-van Eck,W., Dam,W.A., Mulder.N.H., Gietema,J.A., de Vries,E.G. and de Jong,S. (2012) Pro- and anti-apoptotic effects of p53 in cisplatin-treated human testicular cancer are cell context-dependent. *Cell Cycle.* **11,** 4552-4562.

6. Oleaga,C., Welten,S., Belloc,A., Solé,A., Rodriguez,L., Mencia,N., Selga,E., Tapias,A., Noé,V. and Ciudad,C.J. (2012) Identification of novel Sp1 targets involved in proliferation and cancer by functional genomics. *Biochem. Pharmacol.* **84,** 1581-1591.

7. Fung,H. and Demple,B. (2005) A vital role for Ape1/Ref1 protein in repairing spontaneous DNA damage in human cells. *Mol. Cell.* **17,** 463-470.

8. Poletto,M., Lirussi,L., Wilson,D.M.3^rd^ and Tell,G. (2014) Nucleophosmin modulates stability, activity, and nucleolar accumulation of base excision repair proteins. *Mol. Biol. Cell.* **25**, 1641-1652.

9. Khoronenkova,S.V. and Dianov,G.L. (2013) USP7S-dependent inactivation of Mule regulates DNA damage signalling and repair. *Nucleic Acids Res.* **41,** 1750-1756.

10. Poletto,M., Malfatti,M.C., Dorjsuren,D., Scognamiglio,P.L., Marasco,D., Vascotto,C., Jadhav,A., Maloney,D.J., Wilson,D.M.3rd., Simeonov,A., *et al.* (2015) Inhibitors of the apurinic/apyrimidinic endonuclease 1 (APE1)/nucleophosmin (NPM1) interaction that display anti-tumor properties. *Mol. Carcinog.* doi: 10.1002/mc.22313 [Epub ahead of print].

11. Xu,X.M., Tang,J.L., Chen,X., Wang,L.H. and Wu,K.K. (1997) Involvement of two Sp1 elements in basal endothelial prostaglandin H synthase-1 promoter activity. *J. Biol. Chem.* **272,** 6943-6950.
